# Anterior cruciate ligament autograft maturation on sequential postoperative MRI is not correlated with clinical outcome and anterior knee stability

**DOI:** 10.1007/s00167-021-06777-4

**Published:** 2021-11-05

**Authors:** Patricia M. Lutz, Andrea Achtnich, Vincent Schütte, Klaus Woertler, Andreas B. Imhoff, Lukas Willinger

**Affiliations:** 1 Department for Orthopedic Sports Medicine, Technical University Munich, Ismaninger Strasse 22, 81675 Munich, Germany; 2 Department for Orthopedic and Trauma Surgery, Martin-Luther-University Halle-Wittenberg, Ernst-Grube-Strasse 40, 06120 Halle (Saale), Germany; 3 Department of Diagnostic and Interventional Radiology, Technical University of Munich, Ismaninger Strasse 22, 81675 Munich, Germany

**Keywords:** Anterior cruciate ligament, Graft healing, Ligamentization, MRI, Hamstring autograft, Graft maturation

## Abstract

**Purpose:**

Magnetic resonance imaging (MRI) signal intensity is correlated to structural postoperative changes of the anterior cruciate ligament (ACL) autograft. The purpose of this study was to investigate the ACL autograft maturation process via MRI over 2 years postoperatively, compare it to a native ACL signal and correlate the results with clinical outcome, return to preinjury sports levels, and knee laxity measurements.

**Methods:**

ACL autograft signal intensity was measured in 17 male patients (age, 28.3 ± 7.0 years) who underwent ACL reconstruction with hamstring autograft at 6 weeks, 3-, 6-, 12-, and 24 months postoperatively by 3 Tesla MRI. Controls with an intact ACL served as control group (22 males, 8 females; age, 26.7 ± 6.8 years). An ACL/PCL ratio (APR) and ACL/muscle ratio (AMR) was calculated to normalize signals to soft tissue signal. APR and AMR were compared across time and to native ACL signal. Clinical outcome scores (IKDC, Lysholm), return to preinjury sports levels (Tegner activity scale), and knee laxity measurement (KT-1000) were obtained and correlated to APR and AMR at the respective time points.

**Results:**

The APR and AMR of the ACL graft changed significantly from the lowest values at 6 weeks to reach the highest intensity after 6 months (*p* < 0.001). Then, the APR and AMR were significantly different from a native ACL 6 months after surgery (*p* < 0.01) but approached the APR and AMR of the native ACL at 1- and 2 years after surgery (*p* < 0.05). The APR changed significantly during the first 2 years postoperatively in the proximal (*p* < 0.001), mid-substance (*p* < 0.001), and distal (*p* < 0.01) intraarticular portion of the ACL autograft. A hypo-intense ACL MRI signal was associated with return to the preinjury sports level (*p* < 0.05). No correlation was found between ACL MRI graft signal and clinical outcome scores or KT-1000 measurements.

**Conclusion:**

ACL grafts undergo a continuous maturation process in the first 2 years after surgery. The ACL graft signals became hyper-intense 6 months postoperatively and approximated the signal of a native intact ACL at 12- and 24 months. Patients with a hypo-intense ACL graft signal at 2 years follow-up were more likely to return to preinjury sports levels. The results of the present study provide a template for monitoring the normal ACL maturation process via MRI in case of prolonged clinical symptoms. However, subjective outcome and clinical examination of knee laxity remain important to assess the treatment success and to allow to return to sports.

**Level of evidence:**

III.

## Introduction

Anterior cruciate ligament (ACL) reconstruction aims to restore knee stability and regain normal knee function. This allows safe return to activity and reduce the risk of re-injuries. Despite advances in surgical techniques, early ACL re-tears are still a major concern [30, 32, 37]. One reason for early re-ruptures is biological failure due to the remodelling process [29]. The graft maturation process (also known as ligamentization) during the early postoperative phase negatively affects the biomechanical properties of the graft [36, 41, 46]. The decrease of mechanical strength during the first period is of clinical importance as it puts the graft at risk of not meeting its demands [2, 18, 21, 23, 40, 46, 47]. Hence, assessing ACL graft maturity before returning to sports is of high clinical interest and magnetic resonance imaging (MRI) has been shown to be a feasible option [11, 12]. Animal models showed that MRI signal intensity is significantly correlated to structural properties of the ACL graft [5, 46]. A high signal intensity on MRI has been linked to decreased mechanical properties such as low load to failure [46]. In recent years, several MRI studies showed that signal intensity changes of the ACL graft are correlated with the progress of graft maturation in human patients [9, 11, 27, 43]. ACL graft MRI signal intensity increases during the first 6 months followed by a subsequent decline [16, 42, 45, 48]. These findings are in line with histological findings which suggest a high vascularity and cell proliferation process during the early remodelling phase starting after three to nine months postoperatively [1, 29, 33, 34, 44].

However, current studies evaluating the MRI signal changes of ACL autografts and their correlation to clinical outcome and knee laxity remain inconclusive [6, 12, 16, 17, 35]. It is further unknown how ACL autograft MRI signal changes compare to a native ACL MRI signal [11, 12, 16, 17, 24]. Concerning sports activities, reported rates of return to sports after ACL reconstruction (ACL-R) vary in the literature [4, 19, 22, 31]. Previous research stated a low return to preinjury sports levels 1 year after ACL-R [15], whereas a clear increase 2 years postoperatively was described [19, 22]. An additional aspect remains the significantly increased incidence rate of second injuries to the ACL autograft in the first year postoperatively [23].

Therefore, the purpose of this study was to investigate (1) the MRI signal intensity changes of hamstring ACL autografts over the first 2 years postoperatively and compare them to a native ACL and (2) to correlate the MRI signal with clinical outcome scores, return to preinjury sports levels and knee laxity measurements. It was hypothesized that (1) the ACL would mature over time at 1 and 2 years postoperatively and would approximate the signal of a native ACL at final follow-up and (2) a higher grade of graft maturation (hypo-intense MRI signal) would positively correlate with better clinical outcome, return to preinjury sports levels, and knee stability.

## Materials and methods

Ethical approval was obtained from the Ethics Committee of the technical University Munich (329/19S). All procedures performed were in accordance with the ethical standards of the institutional and/or national research committee and with the 1964 Declaration of Helsinki and its later amendments or comparable ethical standards. All patients gave their written informed consent to participate in this investigation.

### Patients

Patients were eligible for inclusion in this study after undergoing unilateral four-strand autograft ACL-R (semitendinosus ± gracilis tendon) following ACL rupture diagnosed by clinical examination and MRI scans and confirmed arthroscopically at time of surgery. Patients were excluded from the study if they had a history of previous surgery at the index knee, a cartilage injury or ligamentous injuries other than ACL rupture including collateral ligaments and posterior cruciate ligament (PCL). Patients with contralateral knee injuries were excluded. Concomitant meniscal lesions were not an exclusion criterion. Baseline demographic variables including age and gender were manually collected using clinical documentation.

30 patients without previous or acute ligamentous knee injuries were included and served as age-matched control group for defining an intact native ACL MRI signal baseline at one time point. Data were collected prospectively in the patient’s cohort but retrospectively compared with the control group.

### Knee laxity measurements

All patients underwent clinical examination of the knee including previously validated KT-1000 arthrometer measurements (MEDmetric, San Diego, CA, USA) by a knee trained orthopaedic surgeon (blinded) at 12- and 24 months after surgery. The KT-1000 arthrometer measurements were performed using a standardized 134 N anterior drawer force at 30° knee flexion and side-to-side differences were recorded.

### Clinical outcomes

Patient-reported clinical outcome scores were measured using the subjective Form of the International Documentation Society Score (IKDC) and the Lysholm score preoperatively and at 6-, 12-, and 24-month postoperatively [13, 39]. Tegner activity scale (TAS) was used to measure postoperative sports participation at 1- and 2- years follow up to determine if a patient achieved the same sports level compared to the pre-injury state. The patient was considered to have returned to the same sports level when preinjury and postoperative TAS revealed the same value.

### Imaging procedures

**Fig. 1:**
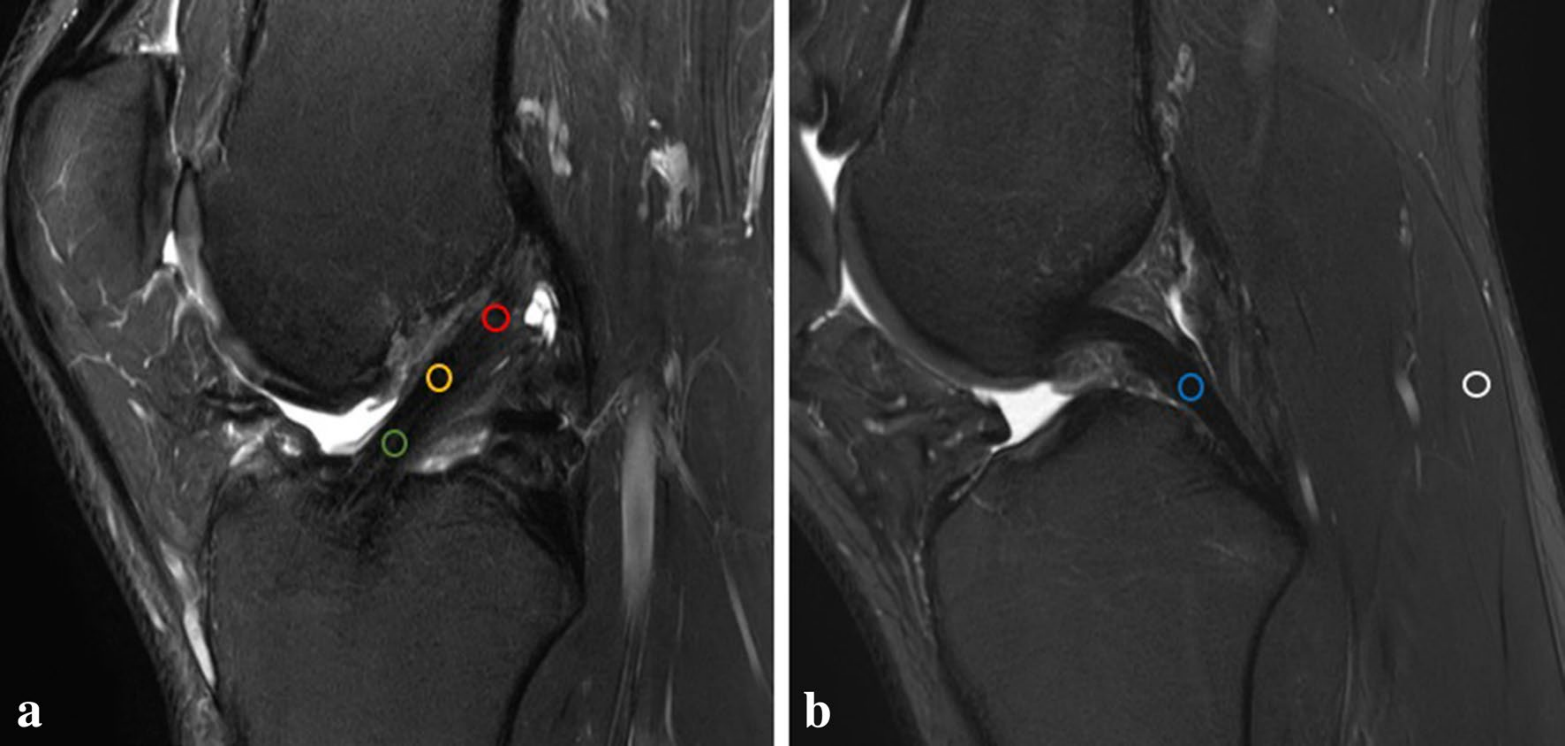
Magnetic resonance imaging signal intensity was measured within a standardized 4 mm diameter circle at the respective region of interest at (**A**) the proximal (red), mid-substance (yellow) and distal (green) portion of the anterior cruciate ligament autograft and (**B**) at the mid-substance of the posterior cruciate ligament (blue) and the medial head of the gastrocnemius (white)

Postoperative MRI examination were performed at 6-weeks, 3-, 6-, 12- and 24- months after ACL reconstruction using a 3 Tesla whole-body scanner (Ingenia, Philips Healthcare, The Netherlands) and a dedicated 8-channel knee coil to continuously monitor the ACL graft maturation process. On sagittal high-resolution proton-density (PD) intermediate-weighted MR images parallel to ACL (repetition time 5000 ms, echo time 45 ms, field of view 160 mm, in-plane resolution 0.4 × 0.4 mm, slice thickness 2.5 mm, acquisition time 4:15 min) the MRI signal intensity was measured manually in five regions of interest (ROI): intraarticular proximal ACL, mid-substance ACL, distal ACL, mid-substance PCL, medial head of the gastrocnemius muscle (Fig. 1). All measurements were performed on a conventional PACS system (Sectra Medical Systems, Sweden). A standardized 4 mm diameter circle was drawn at each ROI and the average MRI signal intensity was automatically calculated by the imaging software [28, 38]. The MRI signal outcome was not affected by contrast and brightness settings. To normalize the signal intensity, an ACL/PCL signal ratio (APR) and an ACL/muscle ratio (AMR) were calculated (each for the proximal, mid-substance, and distal ACL portion) by dividing the ACL signal intensity by the signal of the intact PCL or the medial head of the gastrocnemius muscle. Muscle tissue was chosen as a second internal reference to avoid systematic measurement error caused by variation due to possible degenerative changes of the PCL. We believe that not including the measurement of background noise (proton density of extracorporeal hydrogen atoms) the reliability and accuracy is increased. The APR and AMR measurements were performed independently by two knee trained orthopaedic surgeons (blinded) and the average of both measurements was used for analysis.

### Surgical technique

Arthroscopic ACL-R was performed in single-bundle technique with an autologous hamstring graft (semitendinosus ± gracilis tendon) and anatomic femoral and tibial tunnel placement. The femoral tunnel was drilled via an anteromedial portal according to the diameter of the ACL autograft. A cortical suspension device (ACL TightRope, Arthrex, Naples, USA) was used for femoral graft fixation and a bio-absorbable interference screw (Arthrex, Naples, USA) was used for tibial fixation. Concomitant meniscus lesions were treated by either all-inside meniscus suture devices (Fast-Fix, Smith&Nephew, London, UK) or inside-out technique.

### Postoperative rehabilitation

The postoperative protocol after ACL-R consisted of 6 weeks of partial weight-bearing on crutches with limitation in range of motion (ROM): in the first 6 weeks postoperatively, flexion was only allowed to 90°. After 6 weeks, ROM was no longer limited. A brace (Medi M4, Medi Bayreuth, Germany) was provided for at least 12 weeks. After 8 weeks, sports activities, such as swimming, walking, and cycling and after 12 weeks running were allowed. Return to sport-specific training was allowed after 6 months and full return to contact and/or pivoting sports activities after at least 9 months postoperatively.

### Statistical analysis

Statistical analysis was performed using SPSS software Version 23 (IBM, Armonk, New York, USA) and Microsoft Excel Version 2019 (Microsoft, Redmond, Washington, USA). Descriptive statistics are presented as mean ± standard deviation (SD) for all continuous variables allowing one decimal. Frequencies (*n*, %) were used to obtain descriptive statistics for all categorical variables allowing no decimal. Pearson’s correlation coefficient (*r*) was used to calculated correlations between MRI autograft signal ratios (APR or AMR) and clinical outcomes scores and KT-1000 measurements at the respective time points. The minimal clinically important difference (MCID) for IKDC was set at 9 points, for Lysholm score at 10 points, whereas the minimal detectable change (MDC) for TAS was set at 1 point [7]. Shapiro–Wilk test was used for continuous variables to confirm data normality. Depending on data normality, students *t*-test or Mann–Whitney U test were used to find differences in APR and AMR between ACL grafts and native ACLs. One-way analysis of variance (ANOVA) with Bonferroni correction was used to describe differences in APR and AMR over time. Interclass correlation coefficient (ICC) was calculated for inter-rater reliability between the two readers for APR and AMR measurements. Based on previous research [17], a sample size calculation resulted in a total number of 44 patients with a 2:1 group allocation ratio (29:15 subjects) to detect a difference in APR MRI signal intensity of 30% between ACL autograft and the native ACL at a critical p-value of 0.05 with an actual power of 86.6%.

## Results

Seventeen male patients with a mean age of 28.3 ± 7.0 years who underwent ACL-R were included in the study. All patients were recreational athletes and none of the patients were lost to follow up for the entire study period of 2 years. One patient suffered a contralateral ACL injury 1.5 years after initial ACL-R.

The control group consisted of 30 patients (22 male and 8 female) with an intact ACL. These patients were treated for either meniscal tears, chondral lesions, or patellofemoral instability and had a ligamentous stable knee. The mean age of the control group was 26.7 ± 6.8 years (Table 1).

**Table 1:** Demographic data comparing the patient group with the control group

	Patient group	Control group	*p *value
Age	28.3 ± 7.0	26.7 ± 6.8	n.s
Gender (male/female)	17/0	22/8	*p* = 0.038
BMI (kg/m^2^)	25.3 ± 3.0	23.8 ± 2.7	n.s
Affected side (right/left)	5/12	14/16	n.s
Pre-injury Tegner score	6 (range 4–9)	–	n.a

*BMI* body mass index, *n.s.* not significant, *n.a.* not applicable

The ICC values for reliability of MRI measurements were 0.834 (95% CI 0.776–0878) for APR and 0.871 (95% CI 0.825–0.906) for AMR indicating excellent reliability.

### Native ACL

The ACL/PCL MRI signal ratio (APR) of the native intact ACL was 2.6 ± 1.0 at the proximal portion, 2.6 ± 0.9 at the mid-substance and 3.4 ± 1.4 at the distal portion, meaning that the signal of the native ACL was 2.6–3.4 times more intense than the PCL. The ACL/muscle ratio (AMR) was 0.4 ± 0.2 proximally, 0.4 ± 0.2 at mid-substance and 0.6 ± 0.2 distally, meaning that the native ACL had only 40–57% MRI signal intensity compared with the medial head of the gastrocnemius muscle. The APR and AMR of the distal ACL were significantly higher than the APR and AMR of the proximal and mid-substance ACL (APR *p* < 0.05, AMR *p* < 0.01, see also Fig. 3F).

### APR measurements

**Fig. 2:**
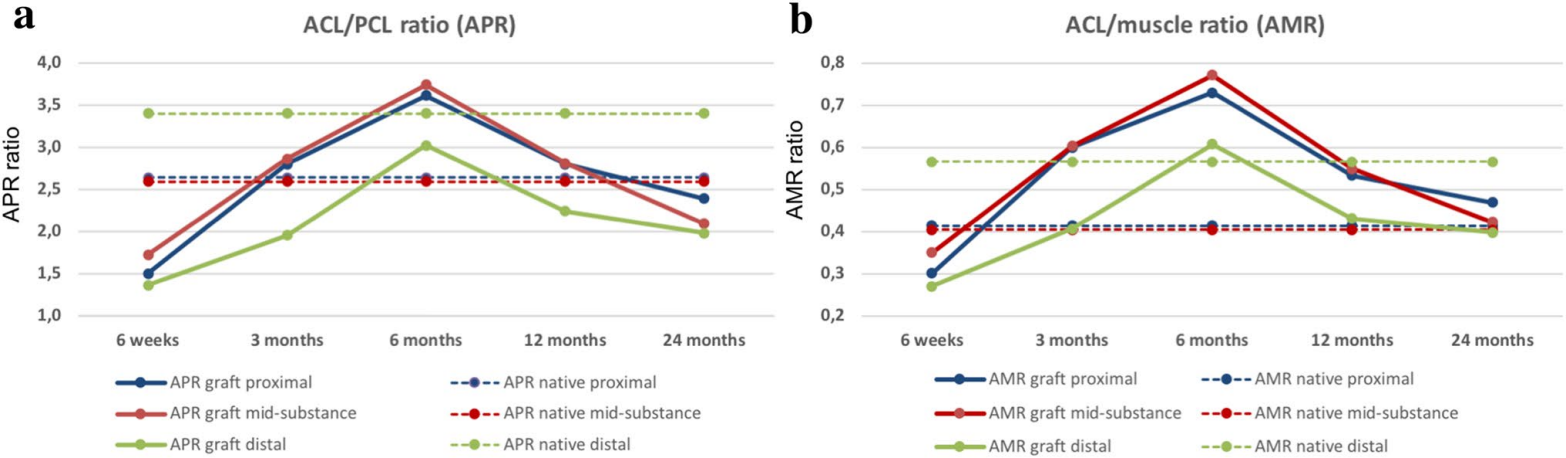
The ACL autograft underwent significant transformation during the first 2 years after ACL reconstruction. This is shown by a significant increase in APR (**A**) and AMR (**B**) from 6 weeks to 6 months after surgery before dropping to reach values of native intact ACL at 1 and 2 years postoperatively. The distal portion of the native ACL exhibits a significantly more hyperintense signal compared to the proximal and mid-substance portion. The distal part of the ACL autograft shows a significantly lower APR and AMR at 3 months compared to the mid-substance

The APR of the ACL autograft over the postoperative period is shown in Fig. 2A. The APR changed significantly during the first two years postoperatively in the proximal (*p* < 0.001), mid-substance (*p* < 0.001), and distal (*p* < 0.01) intraarticular portion of the ACL autograft. The distal ACL portion showed the lowest APR and was significantly different to the mid-substance (*p* < 0.05) ACL graft Sects. 3 months postoperatively. There was no difference in APR between the different measurement locations at 1- and 2 years follow up.

The proximal ACL graft underwent a significant MRI signal change from 6 weeks to 6 months after surgery and showed the highest signal intensity at this time point (*p* < 0.001). The APR subsequently declined to approximate the APR of a native proximal ACL at 1 and 2 years. The proximal APR was significantly lower than a native ACL at 6 weeks (*p* < 0.001) and higher at 6 months (*p* < 0.05) after surgery.

Similarly, the APR of the mid-substance ACL graft ran through a significant transformation from 6 weeks to 2 years postoperatively. While the APR 6 months after ACL-R is significantly different from a native mid-substance ACL (*p* < 0.01), the APR after 1 and 2 years showed a similar MRI signal compared with a native ACL.

APR of the distal section of the ACL graft showed a significant rise within the first 6 months postoperatively (*p* < 0.05) before dropping gradually at 1 and 2 years. The APR of distal section of the ACL autograft was significantly lower than the native ACL at all time points except from 6 months postoperatively (all *p* < 0.01).

### AMR measurements

The AMR of the ACL autograft over the postoperative period is shown in Fig. 2B. The AMR changed significantly at the proximal (*p* < 0.001), mid-substance (*p* < 0.001), and distal (*p* < 0.05) part of the ACL graft.

AMR of the proximal ACL portion rose significantly from 6 weeks to 6 months (*p* < 0.001) after surgery and was by then significantly different to a native ACL (*p* < 0.001). Subsequently, the AMR started to decline to reach a similar AMR of the native proximal ACL at 1 and 2 years.

The mid-substance AMR increased significantly from 6 weeks to 6 months postoperatively (*p* < 0.001) before decreasing at 1 year and 2 years postoperatively (*p* < 0.01). The AMR was significant higher at 3- and 6 months after ACL-R compared with a native ACL (*p* < 0.001) but was not different from a native mid-substance ACL at 1 and 2 years.

**Fig. 3:**
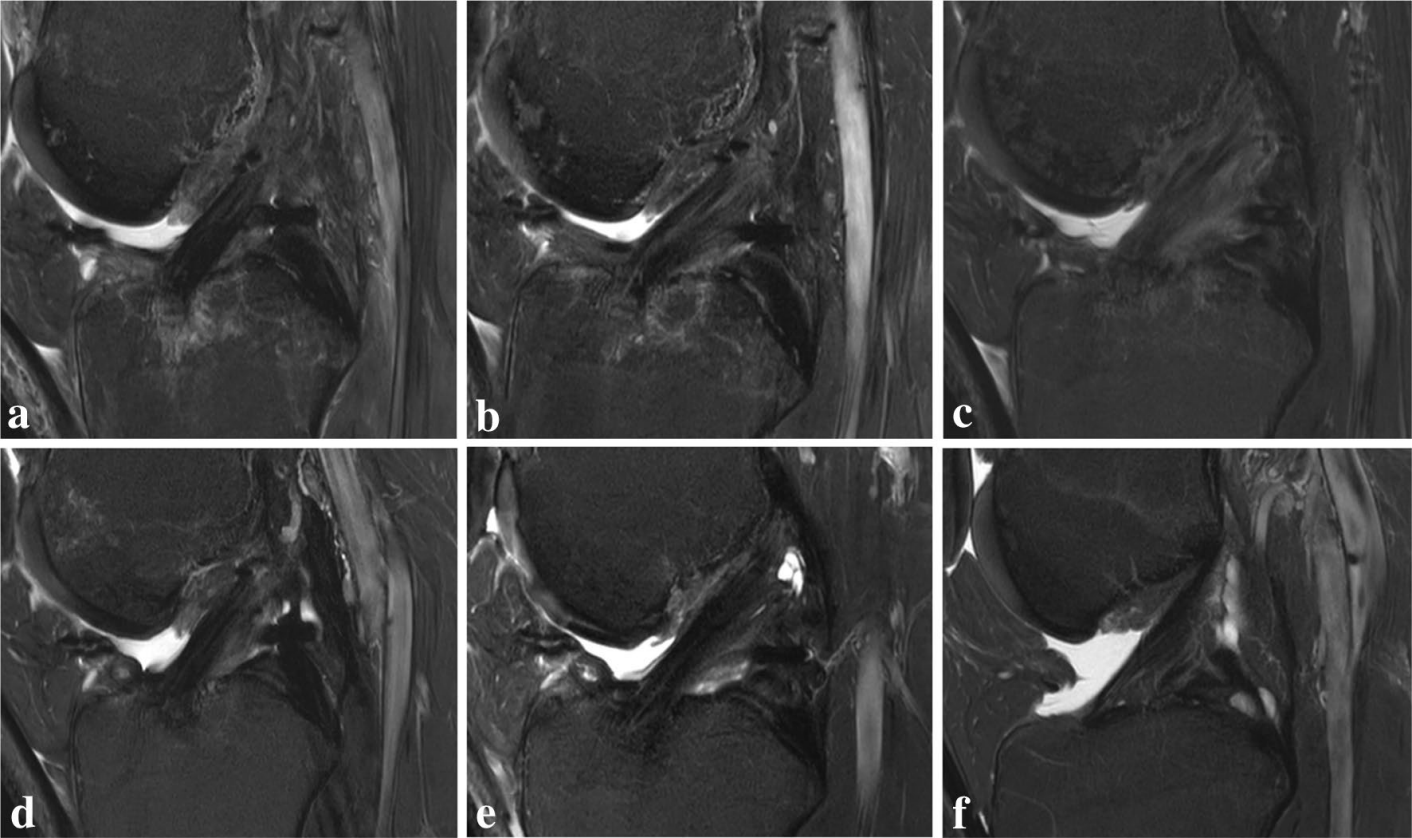
The maturation process of the anterior cruciate ligament autograft exhibits a significant MRI signal change from hypo-intense at (**A**) 6 weeks postoperatively to more hyper-intense at (**B**) 3- and (**C**) 6-months after ACL-R indicating greater disorganization of collagen tissue and higher water content. The MRI signal intensity decreases thereafter at (**D**) 1- and (**E**) 2-years post ACL reconstruction to approximate the signal of a native ACL (**F**). The distal native ACL revealed a more hyper-intense signal compared to the proximal and mid-substance ACL

The distal AMR rose significantly between 6 weeks and 6 months postoperatively (*p* < 0.05). It then dropped slowly to the lowest AMR at 2 years postoperatively. AMR of the distal intraarticular graft section was significantly lower than a native ACL at 2 years after surgery (*p* < 0.05, Fig. 3).

### Correlation to clinical outcome, knee stability, and return to sports

Anterior knee laxity measured by KT-1000 device was 1.9 ± 1.7 mm and 2.4 ± 2.3 mm higher compared to the healthy contralateral knee at 1 and 2 years postoperatively, respectively. For neither APR nor AMR a significant correlation with KT-1000 measurements at the respective time points could be identified.

**Fig. 4:**
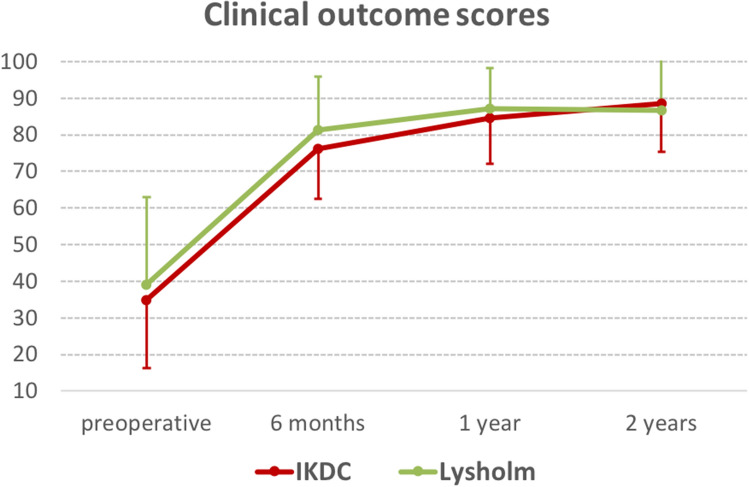
Patient reported outcome scores (Subjective IKDC and Lysholm score) improved significantly from preoperative to 6 months, 1-, and 2 years after ACL reconstruction

IKDC and Lysholm score improved significantly from preoperative values to postoperative values at 6 months, 1-, and 2 years postoperatively (all *p* < 0.001, Fig. 4). There was also a significant increase in clinical outcome scores between 6 months and 1 year postoperatively but not from 1 to 2 years (IKDC: *p* < 0.001, Lysholm score *p* < 0.05). APR and AMR were not associated with IKDC and Lysholm score at any time point.

### Return to preinjury sports levels

94% (16/17) of the patients returned to sports within 2 years after ACL-R. One patient suffered a contralateral ACL injury 1.5 years after initial ACL-R and could therefore not RTS at final follow-up. Median TAS was 6 (range 4–9) before injury, 4 (range 3–6) after 6 months, 5 (range 1–7) after 1 year and 6 (range 1–9) at final follow up. At 1 year and 2 years follow-up, 41% (7/17 patients) and 65% (11/17) achieved the preinjury TAS level, respectively. TAS was not correlated with APR or AMR at any time point within the small patients’ cohort. However, APR and AMR of the ACL mid-substance were significantly lower (hypo-intense) in patients who could return to the preinjury sports level compared to those who did not achieve the same sports level (*p* < 0.05).

## Discussion

The most important findings of our study were that an ACL autograft is undergoing a significant transformation process during the first 2 years postoperatively which is consistent with the previously described ligamentization process [9, 20, 45]. These findings confirmed our first hypothesis that an ACL graft exhibits a high MRI signal intensity 6 months after surgery but approximates the MRI appearance of a native ACL at 1- and 2 years postoperatively. Hypointense MRI signal (lower APR or AMR) did not correlate with higher clinical outcome scores and lower anterior knee laxity what contradicted our second hypothesis. However, patients with lower mid-substance ACL signal intensity were more likely to return to the preinjury sports level.

It is known that T2 relaxation time is correlated to tissue water content and collagen orientation, with higher values indicating greater disorganization and lower values representing less disorganization [5, 9]. After ACL-R, values are expected to increase due to graft remodelling followed by a subsequent reduction [16, 42, 45]. Accordingly, changes in MRI signal of the ACL graft can be observed due to ultrastructural changes of graft over time. Previously, research on ACL autograft maturation used different MRI measurement methods, such as the signal/noise quotient (SNQ) [6, 8, 12, 17, 21, 26, 38, 48], signal intensity ratio (SIR) [11], or T2 mapping [3, 9, 16, 24, 45]. In the present study, the graft signal intensity on MRI was normalized to the subject-specific signal intensity of both the PCL and the gastrocnemius muscle in order to minimize inter-scan variability. Muscle tissue was chosen as a second internal reference to avoid systematic measurement error caused by variation due to possible degenerative changes of the PCL. It was concluded that not including the measurement of background noise (proton density of extracorporeal hydrogen atoms) the reliability and accuracy was increased. This assumption is based on the experience of the two readers, which showed highly variable background noise between subjects and the location of ROI.

ACL grafts underlie a histological healing process that includes initial avascular necrosis, revascularization, resynovialization, and remodelling [2, 10, 14, 29, 47]. Previous research revealed that the revascularization process influences the ACL graft signal intensity in MRI with signal intensity being significantly associated with time from surgery [18, 25, 27, 38]. In line with previous reports, the graft signal intensity in the present study peaks at 6 months, followed by a constant decrease [9, 20, 45]. In agreement with the results of Chu and Williams [9], the present study suggests an approximation of ACL graft signal intensity to the MRI signal of a native ACL 1- and 2 years postoperatively indicating graft maturity.

More specifically, the native ACL and the ACL autografts demonstrated regional differences in MRI signal intensity. It was observed that distal regions of native ACL exhibited higher APR and AMR (hyper-intense signal) compared to proximal or mid-substance regions (Fig. 3F). In contrast, the distal ACL autograft showed lower APR and AMR (hypo-intense signal) compared to the mid-substance in the early healing phase and this phenomenon was also observed in former studies [20, 21, 38, 45]. Tashiro et al. found higher MRI signal in the proximal and mid-substance part of the ACL graft compared to the distal graft at 6 months postoperatively [38]. The authors assumed that a high graft bending angle at the femoral drill hole contributes to increased graft signals due to elevated tension after footstrike [38]. Ma et al. [21] found also the highest MRI signal in the proximal ACL graft and thought that graft length change patterns during flexion, femoral fixation method, and the angle of the femoral tunnel are contributing factors for a longer maturation process proximally. The proximal and mid-substance section of the ACL autograft seems to exhibit a higher collagen disorganization in the remodelling phase compared to the distal part [20, 21, 38], and is, hence, presumably more vulnerable for re-injury.

To avoid early biological failures, some studies tried to find correlations between MRI findings and functional and clinical outcomes lately. In previous reports, MRI- and KT-1000 measurements after ACL-R were either poorly or not correlated [11, 12]. In agreement with the results of Hakozaki et al. [11], no significant correlation between APR or AMR and KT-1000 measurements was identified after 12 or 24 months in the present study. These findings emphasize the importance of a clinical laxity assessment to ensure knee stability before allowing the patient return to sports.

Furthermore, no correlation between APR/AMR and clinical scores could be found in the present study. These results are further strengthened by a study of Li et al. [17], who were not able to show a significant association between ACL autograft SNQ and IKDC, Lysholm score, and TAS. Most patients (94%) of the present study were able to return to sports and 65% of these recreational athletes were able to achieve the same sports level. Interestingly, lower APR/AMR of the ACL mid-substance part increased the chance of returning to the same preinjury sports level 2 years after surgery. This finding is supported by the results of Li et al. [18], who were able to show a significant positive association between TAS and SNQ values in male subjects.

This study has several limitations. Firstly, histologic data are missing. Therefore, the transformation process of a reconstructed ACL in the first 2 years postoperatively was only described on the basis of MRI examinations. Secondly, only males were included in the ACL-R group so the results cannot be transferred for ACL ligamentization in females. Thirdly, even though the number of patients in this study was low, the statistical power of the current study was 0.87. Considering six MRI scans per patient, the number of subjects was intended to be narrowly restricted to minimize the number of scans and costs. Lastly, the measurement methods (APR, AMR) used in this study have not been used before. Nevertheless, the authors believe that by using two different ratios and by not including background noise measurements the drawback was overcome.

The results of the present study provide a template for monitoring the normal ACL maturation process via MRI in case of prolonged clinical symptoms. However, subjective outcome and clinical examination of knee laxity remain important to assess the treatment success and to allow to return to sports.

## Conclusion

ACL grafts undergo a continuous maturation process in the first 2 years after surgery. The ACL graft signals became hyper-intense 6 months postoperatively and approximate the signal of a native intact ACL at 12- and 24 months. ACL autograft signal intensity exhibits significant differences between the mid-substance and distal portion in the early healing phase. Patients with a hypo-intense ACL graft signal at 2 years follow-up were more likely to return to the preoperative sports level.
